# New waves, new variants, old inequity: a continuing COVID-19 crisis

**DOI:** 10.1136/bmjgh-2021-007031

**Published:** 2021-08-12

**Authors:** Senjuti Saha, Arif Mohammad Tanmoy, Afroza Akter Tanni, Sharmistha Goswami, Syed Muktadir Al Sium, Sudipta Saha, Shuborno Islam, Yogesh Hooda, Apurba Rajib Malaker, Ataul Mustufa Anik, Md Saidul Haq, Tasnim Jabin, Md Mobarok Hossain, Nazifa Tabassum, Hafizur Rahman, Md Jibon Hossain, Mohammad Shahidul Islam, Samir K Saha

**Affiliations:** 1 Child Health Research Foundation, Dhaka, Bangladesh; 2 Li Ka Shing Knowledge Institute, St. Michael's Hospital, Unity Health Toronto, Toronto, Ontario, Canada; 3 Division of Cell Biology, Laboratory of Molecular Biology - MRC, Cambridge, UK; 4 Department of Microbiology, Dhaka Shishu Hospital, Dhaka, Bangladesh

**Keywords:** vaccines, COVID-19, immunisation

**Summary box**

- Bangladesh continues to be severely impacted by the COVID-19 pandemic.
- The country has experienced two waves and is currently fighting its third and deadliest wave, driven by the delta variant.
- In the first week of July 2021, cases have risen by 38%.
- With limited vaccine supplies, increasing variants, a population tired of restrictions and an overwhelmed health system, Bangladesh is at a precipice.
- But more concerningly, our plight is not unique - low-income and middle-income countries now contribute to a higher proportion of global COVID-19 cases but have received the minimum number of vaccine doses.
- A vaccine apartheid has left the countries in the Global South reeling from what is now a preventable disease.
- Despite following all of the ‘rules’ set mainly by high-income countries—vying for bilateral deals with countries/companies and contributing to COVAX—Bangladesh finds itself confronting a disastrous third wave as rich countries prepare to drop restrictions related to SARS-CoV-2.
- Here, we, a group of scientists in Bangladesh, use data from a SARS-CoV-2 genomic surveillance study, our lived experiences and historical trends of vaccine access to argue that it is time for low-income and middle-income countries to realise that as long as we are not self-sufficient in vaccine production, this trend will continue.

## Introduction

Bangladesh, a country of 166 million people, continues to be severely impacted by the COVID-19 pandemic. The first three cases of COVID-19 in Bangladesh were detected on 8 March 2020 among a group of travellers. Since then, the country has experienced two waves—in June 2020 and April 2021. In July 2021, less than 3 months after the second wave, Bangladesh is currently fighting its third and by far the deadliest wave. Like most other countries in South Asia, Bangladesh continues to experience waves of infections caused by different variants of concern, precipitated by the massive inequity in the global COVID-19 vaccine distribution. In this commentary, we, a group of scientists in Bangladesh, hope to draw attention to the alarming third wave, and convince global policymakers of the urgency to act now to control the pandemic globally through equitable vaccinations. We use data from the SARS-CoV-2 genomic surveillance study of the Child Health Research Foundation, which has functioned as a SARS-CoV-2 testing and sequencing centre in Dhaka, Bangladesh, since the beginning of the pandemic in the country. We have conducted systematic genomic surveillance since April 2020, for which samples are randomly selected from positive qPCR tests. In addition, we sequence samples when case history suggests possible antigenic escape.

## Successive waves caused by new variants

**Figure 1:**
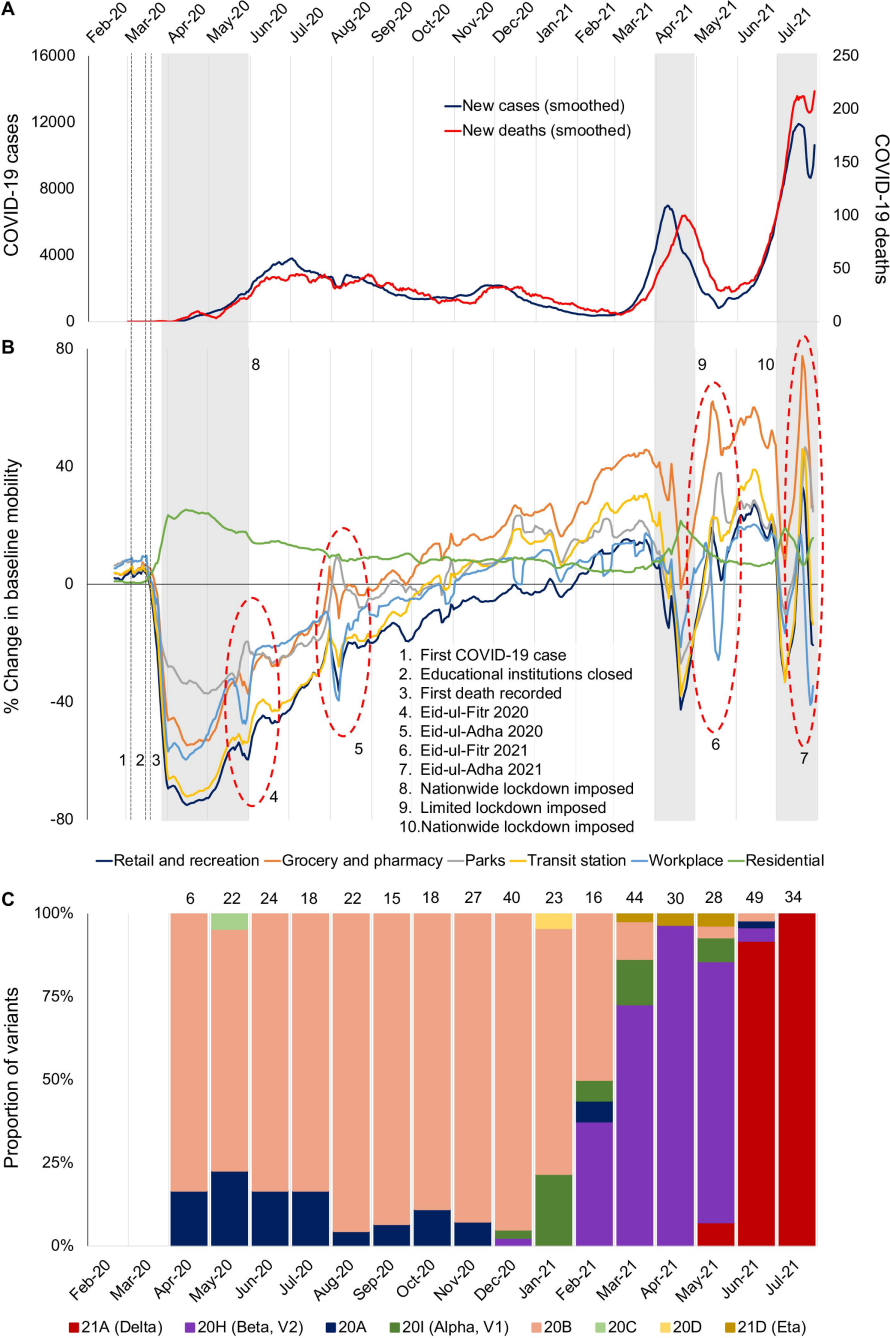
COVID-19 cases, trends in mobility and SARS-CoV-2 variants in Bangladesh, as of 28 July 2021. (A) The 7-day average of confirmed COVID-19 cases and deaths. (B) Mobility of the general population during the COVID-19 pandemic in Bangladesh (Google indicators^2^). (C) Trends of variants circulating in Bangladesh.

In 2020, during the first wave between March and December, cases in Bangladesh rose to a maximum of 3810 cases/day (7-day average) (figure 1A). During that time, test positivity hovered around 20%, suggesting a much higher true incidence of infections.^1^ Non-pharmaceutical interventions including school closures, work from home mandates and flight bans led to reductions in overall mobility, and subsequently to reductions in cases (figure 1B). During this wave, a decline of 25%–70% mobility from baseline was noted in Google mobility indicators even before the institution of restrictions by the government.^2^ Genomic surveillance shows that Nextclade 20B, first observed in Europe in February 2020, dominated the circulating variants during the first wave.^3^ Although importations were observed throughout 2020, most of the sequenced samples belonged to lineages that were imported in March 2020, before the first lockdown and flight restrictions were enacted. By the end of 2020, Bangladesh, along with its neighbour India, was seen as an example of how to control the COVID-19 pandemic. In February 2021, the Government of Bangladesh was congratulated by the Director General of the WHO for its effective efforts in controlling the COVID-19 pandemic.

Despite this initial success, a sharp rise in cases began in early March 2021, leading to the second peak in April 2021 at 7000 cases/day (7-day average), and 24% positivity.^1^ Leveraging the genomic surveillance system in place, we showed that the second wave was driven by the beta variant.^4^ Beta variant was first detected in samples sequenced in December 2020 (1 of 40 sequenced samples, 2.5%) (figure 1C). In February 2021, it constituted 38% (6/16) of all sequenced samples. In March 2021, 73% (32/44) of all sequenced samples were beta, followed by 97% (29/30) in April 2021. Similar to the first wave, introduction of a lineage of the beta variant in November 2020 caused the majority of cases, suggesting that a potential superspreading event was responsible for the country-wide spread.^5^ Leading up to the second wave, mobility indicators bounced back to above baseline, suggesting that the COVID-19 threat was considered less serious by the population after the first wave. In response, the Government of Bangladesh announced a second round of travel restrictions that had a strong impact on mobility but stopped short of a nationwide strict lockdown. By this time, the government had also started the vaccination campaign using Covishield (Oxford-AstraZeneca) vaccines obtained from India through a bilateral agreement that had promised 30 million doses. By mid-May 2021, the daily case numbers had decreased substantially.

Unfortunately, relief following the second wave was short-lived. By the end of May 2021, an increase in COVID-19 cases was seen in districts sharing land borders with India, which was going through a catastrophic second wave driven by the highly transmissible delta variant. The first two cases of the delta variant were detected in our surveillance (2/28, 7%) in May 2021. Although strong border controls and mandatory quarantine for people returning from India were introduced, by June 2021, delta constituted 92% of all samples we sequenced (45/49), and 88% (163/186) of all genomes uploaded to GISAID from Bangladesh. By July 2021, 100% (34/34) of all genomes we sequenced were delta. Cases started rising during the third week of May 2021, coinciding with the rise in the detection of the delta variant.

Given the explosive growth of the delta variant seen in other countries, it is not surprising that by the end of the first week of July, cases per day had risen to 11 525, with a test positivity rate of 31%; numbers of daily cases and deaths have broken every previous record (figure 1A). Hospital beds are quickly filling up, and hospitals in western districts bordering India, where the delta variant first took hold, are running short of medical oxygen. Given the lack of vaccine supplies, and the success of past non-pharmaceutical interventions, the Government of Bangladesh yet again announced a strict national lockdown on 1 July 2021 to control the third wave. However, after more than a year of restrictions and infections, fatigue and reluctance in adhering to non-pharmaceutical interventions may blunt the impact of such interventions. Cases continue to rise and by 28 July cases had risen to 15 192 from 8301 on 1 July. Positivity rate is >30%, and the effects of the lockdowns are yet to be seen.

Bangladesh is at a precipice. With limited vaccine supplies, increasing variants and a population tired of restrictions, it may be running out of options. It is unclear whether the health system of Bangladesh will be able to cope with the current or perhaps the future waves to come. Bangladesh has three beds per 10 000 people,^6^ and the burden of infectious disease in countries like Bangladesh is high even without COVID-19. With resources diverted towards COVID-19, care for other diseases continues to be neglected. For example, diagnosis and treatment of multidrug-resistant tuberculosis have been severely hampered, as has data collection on dengue fever transmission. Indirect impact of COVID-19 on neonatal deaths has been reported,^7^ and impact on maternal health is estimated to be substantial.^8^ There has been a dip in childhood vaccination efforts.^9^ Rise in indiscriminate use of antimicrobials is paving the path for further rise of antimicrobial-resistant bacteria. All these factors may combine to erode the decades of progress Bangladesh has made in improving health outcomes. Controlling the pandemic through vaccinations is of urgent importance.

## Global vaccine inequity prevents effective control of the pandemic

In Bangladesh, <5% of the population has been partially/fully vaccinated as of 28 July 2021.^10^ Known for its strong track record in vaccination policies and campaigns,^11^ and high vaccine confidence,^12^ Bangladesh started laying the groundwork for vaccine access when the earliest clinical trials started. This included making contributions to COVAX and making deals with the Serum Institute of India. However, India’s ban on vaccine exports to control their own outbreak has been a massive blow to Bangladesh and many other low-income and middle-income countries (LMICs) that were dependent on the supply of the Covishield/AstraZeneca vaccine from the Serum Institute of India. This example of vaccine nationalism is not a one-off case, and follows the path set by high-income countries who brokered exclusive deals with manufacturers and monopolised supply chains to have enough quantities to vaccinate their populations many times over. The Government of Bangladesh has recently promised to accelerate the country’s vaccination drive. USA donated about 5.5 million doses of Moderna vaccine, and 6 million Pfizer doses are expected to be received under COVAX. Bangladesh is paying for vaccines from Russia and China for doses of the Sputnik and Sinopharm vaccines. Although there is some supply coming in, these represent a slow drip compared with the massive need, and uncertainties persist until all doses physically reach the country. Unpredictability of supply makes planning difficult, and frustrations and confusions about immunisation dates, doses and brands have provided opportunities for misinformation and rumours to spread.

The plight of Bangladesh is not unique. Many countries in Asia, sub-Saharan Africa and Latin America now contribute a large proportion of global COVID-19 cases and will likely continue to fight incessant cycles of infections. And with high rates of infections, new variants continue to emerge (eg, gamma in Brazil and delta in India), which in turn are beginning to affect high-income countries as well.^13^ The delta variant has now spread to at least 100 countries, and has become the most prevalent SARS-CoV-2 variant. The only solution going forward is vaccinating populations across all countries as quickly as possible to bring down global case numbers and deaths. Unfortunately, treating vaccines as a private good being snatched up by the richest countries has established a vaccine apartheid that has left countries in the Global South reeling from what is now a preventable disease.

## Dismantling the vaccine apartheid

COVID-19 is unlikely to be eliminated globally anytime soon, and regular and mass vaccination may be the only way forward to keep mortality and morbidity low. This requires equity in access to treatments, preventions and protecting vulnerable populations irrespective of country borders. As of 27 July 2021, 3.93 billion doses of COVID-19 vaccines have been administered; 27.5% of the world’s population has received a COVID-19 vaccine (at least one dose), but only 1.1% of people in low-income countries have been partially or fully vaccinated.^14^

The SARS-CoV-2 pandemic is new, but the inequity in access to life-saving vaccines is not. In 2009, Bangladesh introduced the *Haemophilus influenzae* type b (Hib) vaccine in the national immunisation programme to reduce child morbidity and mortality due to meningitis and pneumonia.^15^ While now considered a huge success, as this vaccine is estimated to prevent at least 3100 deaths every year of <1 year children, it took 21 years for this vaccine to be introduced in Bangladesh since its licensure in the USA. The underlying reasons were two-fold—lack of evidence of disease burden (stemming from lack of surveillance and laboratory resources) and global neglect. Twelve years after the introduction of the Hib vaccine, the massive inequity in access to COVID-19 vaccines (despite evidence of disease) shows us that this pandemic is only a mirror of the age-old, colonial inequity in access to essential medicines.

The ‘we are all in this together’ rhetoric and mechanisms like COVAX from the early days of the pandemic suggested that the approach to this disease may have been different. Bangladesh essentially followed all of the ‘rules’ set mainly by high-income countries—respecting intellectual property rights, vying for bilateral deals with countries, or with companies, and contributing to COVAX. Yet, it, along with most other Global South countries, finds itself confronting a disastrous third wave as rich countries prepare to declare ‘independence’ from the virus and drop most non-pharmaceutical interventions. Unfortunately, it seems that the rules were never meant to value the lives of all equally.

## Conclusion

Confronting the COVID-19 crisis requires scaling up of global vaccine production through waiving of intellectual property rights and global investment in manufacturing capacity and technology transfer. Considering that such inequities in health have existed since colonial times, it is perhaps time for the Global South to realize that if we are not independent, this trend will continue. LMICs need to start working together to build the technological know-how of vaccine production, and democratize it globally, so that we can respond quickly at any place and at any time, especially to any new and more dangerous variants that might emerge. Vaccine access for LMICs should not be an afterthought, and neither should countries like Bangladesh continue to depend on donations. This cycle of inequity in access to vaccines needs to end, and it requires strong efforts from both the Global South and the North. As populations in rich countries get back to enjoying sporting events and vacations, it is important to note that last year has taught us that nobody is safe until everybody is safe. We are in this crisis together, whether we act on it together or not.
